# Cytomegalovirus infection in patients receiving bispecific antibodies for multiple myeloma and B-cell malignancies: a single-center cohort and meta-analysis

**DOI:** 10.3389/fonc.2026.1918863

**Published:** 2026-08-26

**Authors:** Eduardo Aparicio-Minguijón, Isabel Rodríguez-Goncer, Nieves López-Muñoz, María Asunción Pérez-Jacoiste Asín, Guillermo Bartolomé Herguedas, Jorge Boán, Adolfo Jesús Sáez Marín, José María Sánchez-Pina, Joaquín Martínez-López, María Calbacho, José María Aguado, Mario Fernández-Ruiz

**Affiliations:** 1 Unit of Infectious Diseases, Hospital Universitario “12 de Octubre”, Instituto de Investigación Sanitaria Hospital “12 de Octubre” (imas12), Madrid, Spain; 2 Department of Medicine, School of Medicine, Universidad Complutense, Madrid, Spain; 3 Centro de Investigación Biomédica en Red de Enfermedades Infecciosas (CIBERINFEC), Instituto de Salud Carlos III, Madrid, Spain; 4 Department of Hematology, Hospital Universitario “12 de Octubre”, Instituto de Investigación Sanitaria Hospital “12 de Octubre” (imas12), Madrid, Spain; 5 Internal Medicine Department, Hospital Universitario “12 de Octubre”, Madrid, Spain

**Keywords:** acute lymphoblastic leukemia, bispecific antibody, cytomegalovirus, lymphoma, meta-analysis, multiple myeloma

## Abstract

**Background:**

Bispecific antibodies (BsAbs) are transformative therapies for multiple myeloma (MM) and B-cell malignancies. Cytomegalovirus (CMV) infection has been reported as a potential complication of unclear clinical magnitude.

**Methods:**

We conducted a retrospective study of MM patients treated with BsAbs at our institution (2020–2025), alongside a systematic review and meta-analysis of clinical trials and observational studies evaluating BsAbs for MM, B-cell lymphoma, and acute lymphoblastic leukemia. Study outcomes included clinically significant CMV infection (csCMVi), any CMV DNAemia, and CMV disease.

**Results:**

Our cohort included 98 BsAb therapy courses (74.5% anti-B-cell maturation antigen [BCMA]) with a median follow-up of 10.4 months. Cumulative incidence rates for csCMVi and CMV disease were 9.2% (9/98) and 3.1% (3/98), respectively. Factors associated with csCMVi at the univariable level were poorer functional status, prior allogeneic hematopoietic stem cell transplantation (allo-HSCT), grade 4 neutropenia, and grade ≥3 cytokine release syndrome (CRS). The meta-analysis (23 studies plus our single-center cohort comprising 2,956 BsAb courses) revealed a pooled cumulative incidence rates of 9% (95% confidence interval [CI]: 4–15%; I^2^ 96.11%) for csCMVi and 1% (95% CI: 0–2%; I^2^ 69.78%) for CMV disease. Incidence was notably higher in studies applying routine CMV DNAemia monitoring and with anti-BCMA agents.

**Conclusion:**

The occurrence of csCMVi or CMV disease during the course of BsAb therapy for MM or B-cell malignancies is uncommon, arguing against routine CMV DNAemia monitoring or antiviral prophylaxis. Prevention approaches, however, may be warranted in patients receiving anti-BCMA BsAbs with additional risk factors such as allo-HSCT or severe CRS.

**Systematic Review Registration:**

https://www.crd.york.ac.uk/prospero/, identifier CRD420251162657.

## 1 Introduction

The paradigm for the treatment of hematologic malignancies has changed with the advent of bispecific antibodies (BsAbs), which are designed to enable simultaneous binding of the CD3 receptor on T cells and a tumor antigen (1). Since the approval of blinatumomab in 2014 for the treatment of B-cell precursor acute lymphoblastic leukemia (B-ALL), the armamentarium has been expanded with the introduction of BsAbs targeting CD20 for B-cell lymphomas, and B-cell maturation antigen (BCMA), G protein-coupled receptor class C group 5 member D (GPRC5D), and Fc receptor-homolog 5 (FcRH5) for multiple myeloma (MM) (2).

Patients receiving BsAbs may be immunocompromised due to their underlying malignancy and the cumulative effect of previous chemotherapy lines and allogeneic hematopoietic stem-cell transplantation (allo-HSCT). In addition, BsAbs cause B-cell aplasia with depletion of plasma cells, which results in hypogammaglobulinemia (3). The sustained T-cell stimulation can lead to cellular exhaustion and functional impairment (4). Finally, and similarly to chimeric antigen receptor T-cell (CAR-T) therapy, cytokine release syndrome (CRS) and immune cell-associated neurotoxicity syndrome (ICANS) are common immune-related adverse effects associated with BsAbs. The management of these toxicities often requires the administration of corticosteroid boluses or anti-interleukin (IL)-6 or anti-IL-1 agents (5).

Cytomegalovirus (CMV) reactivation is a well-established complication of CAR-T therapy, with cumulative incidence rates of CMV infection and end-organ disease estimated at 24.7% and 1.73%, respectively (6). By comparison, the epidemiology and clinical significance of CMV infection in patients receiving BsAbs remain poorly defined. The occurrence of CMV events appears to be uncommon with BsAbs for B-cell lymphoma or B-ALL (7). For instance, no cases of CMV infection were reported in randomized clinical trials (RCTs) of blinatumomab as monotherapy, whereas pivotal trials of anti-CD20 BsAbs have shown low rates of CMV reactivation (8–12). In contrast, BsAbs for MM have been associated with a higher—albeit variable—risk, particularly with anti-BCMA agents (teclistamab, elranatamab, and linvoseltamab). The considerable variability in the rates reported across RCTs and observational studies makes it difficult to determine the true magnitude of this complication (3, 5, 13–16).

Defining the optimal strategies for the prevention and management of CMV infection in the growing population of hematological patients receiving BsAbs constitutes an unmet need. Therefore, we conducted a single-center observational study to investigate the incidence, clinical spectrum, and risk factors for CMV events associated with anti-BCMA, anti-GPRC5D, and anti-FcRH5 BsAbs in MM patients. To gain further insight into this complication, we also performed a systematic review and meta-analysis of CMV infection across the entire spectrum of approved BsAbs for B-ALL, B-cell lymphoma, and MM.

## 2 Methods

### 2.1 Single-center cohort

#### 2.1.1 Study design and patient population

We conducted an observational study including all consecutive MM patients receiving BsAbs at our institution from January 2020 to May 2025. Patients were identified from a prospectively maintained registry of hematologic malignancies. All patients were enrolled at the initiation of BsAbs therapy (day 0). CMV events were recorded from day 0 until 1 month after the discontinuation of the BsAb, unless the patient initiated a subsequent line of therapy or died before completion of the 1-month follow-up period. In the absence of suggestive clinical manifestations, CMV DNAemia was not routinely monitored but tested according to the criteria of the attending clinician.

The study was performed in accordance with the ethical standards laid down in the Declaration of Helsinki and was approved by the local clinical ethics committee.

#### 2.1.2 Study outcomes

We assessed as primary outcomes the occurrence of clinically significant CMV infection (csCMVi), any CMV infection, and CMV disease within the overall number of BsAb courses administered. As secondary aim, we investigated the risk factors for the occurrence of csCMVi.

#### 2.1.3 Study definitions

A *BsAbs course* was defined as continuous treatment with a specific BsAb, encompassing the period from day 0 until 1 month after discontinuation of that agent or initiation of a subsequent line of therapy, whichever occurred first. The characteristics of MM and the scoring system were defined according to the International Myeloma Working Group (IMWG) consensus (17). Functional status was determined by the Eastern Cooperative Oncology Group (ECOG) scale (18). *CMV DNAemia* was defined as CMV DNA detection by quantitative polymerase chain reaction (qPCR) on peripheral blood samples. The occurrence of “blips” in patients receiving letermovir prophylaxis were excluded. *csCMVi* was defined as either the occurrence of CMV disease or asymptomatic CMV infection prompting the initiation of preemptive therapy. In our institution, the CMV DNAemia threshold for initiating antiviral therapy was not formally established and the decision was taken on an individual basis. *CMV disease* included end-organ disease and viral syndrome, as detailed in **Supplementary Methods** (19). To qualify as viral syndrome, alternative causes of acute febrile episodes should have been reasonably ruled out. Additional study definitions, details on the management of CRS and ICANS, and antimicrobial prophylaxis practices are provided as **Supplementary Methods**.

#### 2.1.4 Statistical analysis

Quantitative variables were shown as mean ± standard deviation (SD) or median with the interquartile range (IQR). Qualitative variables were expressed as absolute and relative frequencies. Normality was explored using the Shapiro–Wilk test. Categorical variables were compared using the χ^2^ test or Fisher’s exact test, whereas Student *t* test or Mann–Whitney *U* test were applied for continuous variables. Incidence rates (IRs) with 95% confidence intervals (CIs) were calculated as episodes per BsAb courses-year. Cumulative incidence was calculated as the proportion of BsAb courses with at least one episode of the event of interest divided by the total number of courses. Factors associated with the occurrence of csCMVi were analyzed using Cox regression models for recurrent events. Due to the limited number of events, no multivariable analysis was performed. Unadjusted associations were expressed as hazard ratios (HRs) with the corresponding 95% CIs. Statistical analysis was performed using Stata^®^ version 19.5 (StataCorp, College Station, TX).

### 2.2 Systematic literature review and meta-analysis

#### 2.2.1 Study design

We conducted a systematic literature review and meta-analysis to estimate the incidence of CMV infection across the entire spectrum of patients with hematological malignancies treated with BsAbs. This meta-analysis was designed in accordance with the Preferred Reporting Items for Systematic Reviews and Meta-Analysis (PRISMA) guidelines (20). The protocol was registered in the Prospective Register of Systematic Reviews (PROSPERO) database (CRD420251162657).

#### 2.2.2 Eligibility criteria

We selected RCTs and observational studies (either comparative or non-comparative) including adult and pediatric patients receiving BsAbs for B-ALL, B-cell lymphoma, or MM. Only studies providing data on infectious episodes at both the syndrome and pathogen levels were selected. Reviews, previous meta-analyses, clinical guidelines, case reports, and series with less than 10 patients, editorials, and animal studies were excluded, as were studies lacking essential data (i.e., the overall number of patients and the number of participants that developed infection). All the studies had to be published full-text in English to be considered. In case of partially overlapping publications, only the study with the highest number of patients was included in the analysis.

#### 2.2.3 Search strategy

PubMed (Medline), Embase, and Web of Science databases were searched from inception to September 23, 2025. The complete search strategy is available as **Supplementary Methods**. The references of the resulting articles as well as excluded recent reviews and meta-analysis were reviewed to identify potentially relevant missed publications.

#### 2.2.4 Data extraction

The following data were extracted from each study by two investigators independently (E.A.M. and M.F.R.): study characteristics (year of publication, first author, study design, sample size, and follow-up); hematological condition; characteristics of BsAb therapy; use of antiviral prophylaxis against other herpesviruses such as varicella-zoster virus (VZV); prevention strategy against CMV (preventive therapy or antiviral prophylaxis); definitions used for CMV infection; and outcomes (absolute number of episodes of CMV DNAemia, CMV disease, or csCMVi). When possible, CMV events were classified as CMV DNAemia, disease, and csCMVi according to the definitions detailed above (19). If the analyzed study did not apply these definitions, any reported episode of CMV infection was considered as CMV DNAemia, and any episode of CMV infection grade ≥3 was considered as csCMVi. CMV disease was only adjudicated if an explicit definition of the clinical syndrome was provided. The severity was established according to the applicable version of the Common Terminology Criteria for Adverse Events (CTCAE) (**Supplementary Methods**).

#### 2.2.5 Methodological quality of the included studies

The Newcastle–Ottawa Quality Assessment Scale (NOS) was used to assess the quality of observational cohort studies (21). Studies scoring ≥5 stars were considered to be of moderate to high quality. Discrepancies regarding study eligibility, data extraction, or quality assessment were resolved by consensus.

#### 2.2.6 Outcomes

The study outcome was the cumulative incidence of csCMVi, any CMV DNAemia, and CMV disease in patients receiving BsAbs for hematologic malignancies. We planned a set of sensitivity analyses according to the underlying disease, frequency of monitoring for CMV DNAemia, and type of BsAbs.

#### 2.2.7 Statistical analysis

We performed a meta-analysis of proportions to estimate the pooled cumulative incidence of study outcomes with the corresponding 95% CIs. As most studies did not provide disaggregated data at the treatment-course level (i.e., number of BsAbs therapy courses with at least one episode of CMV infection relative to the total number of BsAbs therapy courses), we used the total number of events as the numerator for all studies, including our single-center cohort (i.e., the number of CMV infection relative to the total number of BsAb courses). Heterogeneity was evaluated by the Cochran’s Q test (which was considered significant at a *P*-value <0.05) and quantified with the I^2^ statistic (22). A random-effects model with the Mantel–Haenszel method was used for pooling results from primary studies. Publication bias was evaluated with funnel plot and Egger test. Statistical analysis and figures were performed with Stata^®^ version 19.5.

## 3 Results

### 3.1 Single-center cohort

#### 3.1.1 Patients’ characteristics

We included 92 MM patients that received 98 BsAb courses, with a median time at risk of 10.4 months (IQR: 2.8 – 18.6). Anti-BCMA BsAbs were the most common agents [74.5% (73/98)], followed by the anti-GPRC5D agent talquetamab [23.4% (23/98)]. Demographics and clinical characteristics are detailed in **Table 1**.

**Table 1:** Demographic and clinical characteristics of 98 courses of BsAbs therapy in the single-center cohort.

Variables	Total cohort
Age, years [mean ± SD]	63.3 ± 10.7
Gender, male [n (%)]	56 (57.1)
MM revised international staging system [n (%)]	
1 pt	22 (30.1)
2 pt	35 (48.0)
3 pt	16 (21.9)
Number of prior lines of therapies [median (IQR)]	3 (2–5)
Prior lines of treatment [n (%)]	
Proteasome inhibitor	96 (98.0)
Immunomodulatory agent	96 (98.0)
Anti-CD38 monoclonal antibody	82 (83.7)
Antibody–drug conjugates	9 (9.2)
Other BsAbs	8 (8.2)
HSCT [n (%)]	82 (83.7)
Autologous	77 (78.6)
Allogeneic	5 (5.1)
CAR-T therapy [n (%)]	6 (6.1)
Positive CMV IgG serostatus at baseline [n (%)]^a^	42/47 (89.4)
Type of BsAbs [n (%)]	
Anti-BCMA	73 (74.5)
Anti-GPRC5D	23 (23.4)
Anti-FcRH5	1 (1.2)
Combination (anti-BCMA plus anti-GPRC5D)	1 (1.2)
Antiviral prophylaxis [n (%)]	
Acyclovir	96 (98.0)
Letermovir (secondary prophylaxis)	1 (1.2)
Immunoglobulin replacement therapy [n (%)]	57 (58.2)
Follow-up period, months [median (IQR)]	10.4 (2.8–18.6)
CRS [n (%)]	68 (69.4)
ICANS [n (%)]	5 (5.1)
CRS/ICANS treatment [n (%)]	
Steroids	20 (20.4)
Tocilizumab	41 (41.8)
Anakinra	3 (3.1)
Grade 4 neutropenia at initiation of BsAb therapy [n (%)]	1 (1.0)
Grade 4 lymphopenia at initiation of BsAb therapy [n (%)]	5 (5.1)
Nadir laboratory values during BsAb therapy [n (%)]	
Hypogammaglobulinemia (IgG <400 mg/dL)	81 (85.3)
Grade 4 neutropenia	40 (41.0)
Grade 4 lymphopenia	61 (61.2)
Discontinuation of BsAb therapy [n (%)]	49 (50.0)
MM relapse [n (%)]	32 (32.7)
All-cause mortality [n (%)]	13 (14.1)

BCMA, B-cell maturation antigen; BsAbs, bispecific antibodies; CAR-T, chimeric antigen receptor T-cell; CMV, cytomegalovirus; CRS, cytokine release syndrome; FcRH5, Fc receptor-homolog 5; GPRC5D, G protein-coupled receptor class C group 5 member D; HSCT, hematopoietic stem-cell transplantation; ICANS, immune effector cell-associated neurotoxicity syndrome; IQR, interquartile rank; MM, multiple myeloma; SD, standard deviation.

^a^ Baseline CMV serostatus before the initiations of BsAbs therapy was available in 47 patients.

#### 3.1.2 CMV events

At least one episode of CMV reactivation prior to the initiation of BsAb therapy occurred in 9.8% (9/92) of patients. CMV DNAemia was determined at least once in 42.9% (42/98) of BsAbs courses. Overall, there were 22 episodes of CMV infection (18 episodes of asymptomatic CMV DNAemia and 4 episodes of disease). The clinical forms of CMV disease included retinitis (two separate episodes in the same patient), hepatitis, and adrenalitis (one episode each). All these cases fulfilled the diagnostic criteria for probable end-organ disease (19), with no episodes of proven disease. Cumulative incidence rates of CMV DNAemia and disease were 13.3% (13/98) and 3.1% (3/98), respectively. Within the episodes of asymptomatic CMV infection, 38.9% (7/18) required preemptive antiviral therapy. The resulting cumulative incidence rate of csCMVi was 9.2% (9/98). The IR of csCMVi was 8.67 episodes/100 person-year (95% CI: 3.97–16.46), whereas the IR of CMV disease was 3.85 episodes/100 person-year (95% CI: 1.05–9.87). The attributable mortality of CMV disease was 25.0% (1/4). Most episodes [83.3% (15/18)] of asymptomatic CMV DNAemia and all the four episodes of CMV disease occurred during the courses of anti-BCMA therapy (**Table 2**). Patients experiencing csCMVi had higher all-cause mortality than those who remained free of this complication [44.4% (4/9) versus 10.8% (9/83), respectively; *P*-value = 0.006]. In contrast, BsAb treatment courses complicated by csCMVi were associated with a lower cumulative incidence rate of MM relapse than those without csCMVi [0.0% (0/9) versus 38.6% (32/89); *P*-value = 0.029).]

**Table 2:** Incidence, clinical characteristics, and viral kinetics parameters of CMV events in the single-center cohort.

*Asymptomatic CMV infection*	n = 18^a^
Number of BsAb courses with at least one episode	13/98
Cumulative incidence (% [95% CI])	13.3 (7.3–21.6)
Number of patients with at least one episode	11/92
Interval from BsAb initiation to the first episode, days [median (IQR)]	99.0 (42.0–235.0)
Requirement for preemptive therapy [n (%)]	7/18 (38.9)
With peak CMV DNAemia <1,000 IU/mL	0/7 (0.0)
With peak CMV DNAemia ≥1,000	7/7 (100.0)
Patients with recurrent infection [n (%)]^b^	4/11 (36.4)
Peak CMV DNAemia, IU/mL [median (IQR)]	1,680 (871–4,408)
*CMV disease*	n = 4
Number of BsAbs courses with at least one episode	3/98
Cumulative incidence (% [95% CI])	3.1 (0.6–8.7)
Number of patients with at least one episode	3/92
Interval from BsAb initiation to the first episode, days [median (IQR)]	81.5 (53.5–115.0)
Clinical syndrome [n (%)]	
Probable retinitis^c^	2/4 (50.0)
Probable hepatitis	1/4 (25.0)
Probable adrenalitis	1/4 (25.0)

BsAbs, bispecific antibodies; CI, confidence interval; CMV, cytomegalovirus; IQR, interquartile range.

^a^ Includes three blips occurring during letermovir prophylaxis.

^b^ At least two episodes separated by both a minimum 2-week interval and at least one negative sample for CMV DNAemia.

^c^ Includes two episodes of retinitis in the same patient.

#### 3.1.3 Predictors of csCMVi

Factors associated with the occurrence of csCMVi at the univariable level were poorer ECOG status (HR: 2.79; 95% CI: 1.54–5.07; *P-*value = 0.004), previous allo-HSCT (HR: 12.16; 95% CI: 1.71–86.45; *P*-value = 0.024), grade 4 neutropenia at the initiation of BsAbs therapy (HR: 20.0; 95% CI: 3.65–109.44; *P*-value 0.007), and grade ≥3 CRS (HR: 19.9; 95% CI: 3.65–109.44; *P*-value = 0.006) (**Supplementary Table 1**). Given the small number of csCMVi events included (n = 9), which precluded any multivariable analysis, these findings should be considered hypothesis-generating only.

### 3.2 Literature review and meta-analysis

#### 3.2.1 Results of literature search and study selection

**Figure 1:**
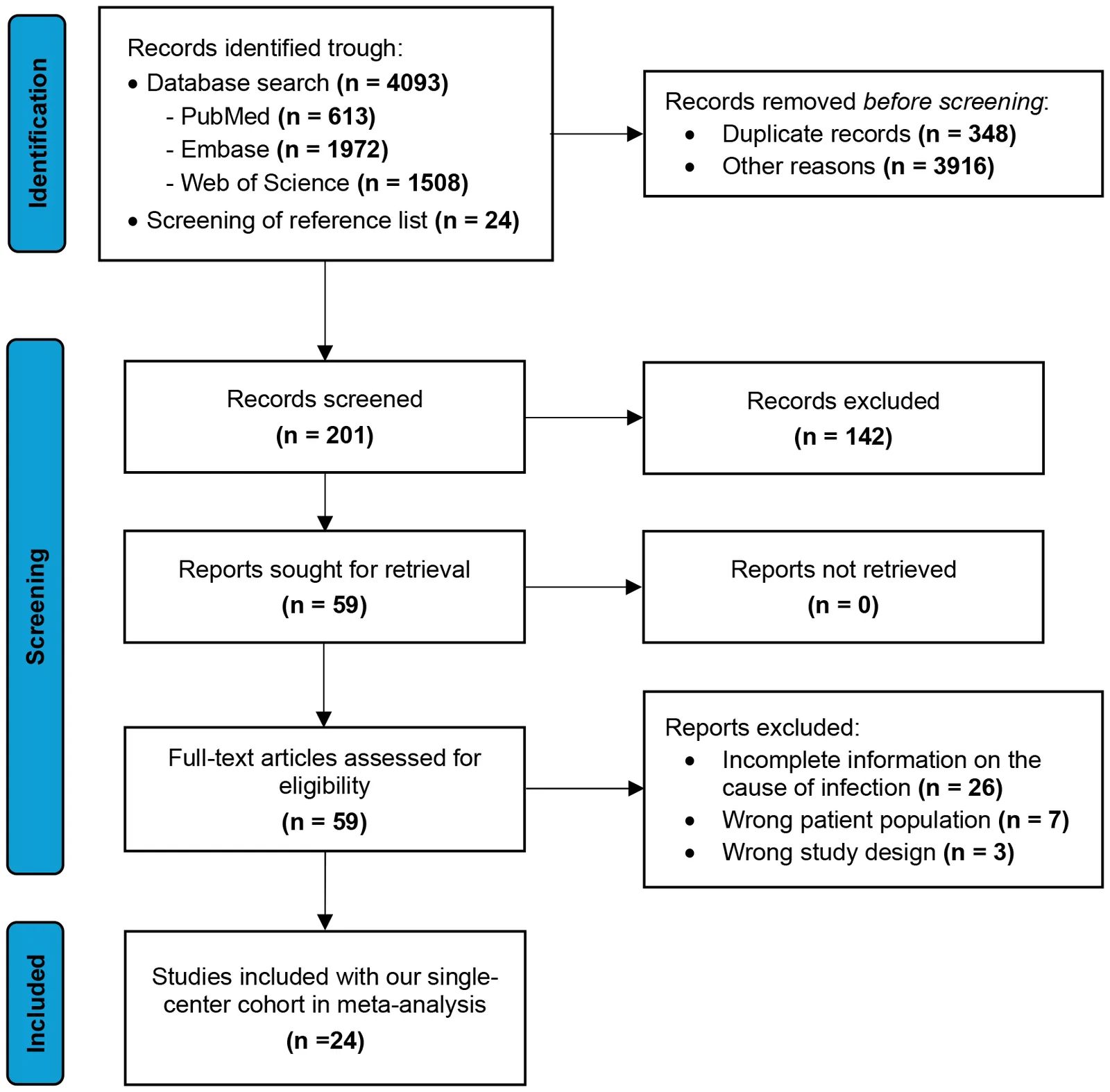
PRISMA flowchart for literature search and study selection.

Our initial search revealed 4,117 articles, 59 of which were full-text evaluated for inclusion. A total of 23 articles were ultimately selected and combined with the present single-center cohort, yielding a total of 24 studies for further analysis (**Figure 1**).

#### 3.2.2 Study and patients’ characteristics

Most studies were RCTs [50.0% (12/24)] (8–11, 13–16, 23–26), including one *post-hoc* analysis [4.2% (1/24)] (16) and one phase 1 first-in-human study [4.2% (1/24)] (23). The remaining 12 studies were observational [50.0% (12/24)], comprising 1 prospective cohort [4.2% (1/24)] (27) and 11 retrospective cohorts [45.8% (11/24)] (3, 5, 27–35) (**Table 3**).

**Table 3:** Description of the studies included in the meta-analysis.

Study	Author and year	Type of study	Underlying malignancy	BsAb therapy	Sample size	Median follow-up (months)
1	Park (2025) (28)	Observational, retrospective	MM	Anti-BCMA (anti-BCMA [54%], combined therapy with anti-CD38 mAb and BsAb or dual BsAb [49%])	61	7.8 (95% CI: 5.9–12.8)
2	Bumma (2024) (13)	RCT	MM	Anti-BCMA (linvoseltamab)	221	200 mg dosing: 14.3 (range: 0.2–38.4); 50 mg dosing: 7.4 (range: 0.4–42.0)
3	Nath (2024) (30)	Observational, retrospective	MM	Anti-BCMA	55	4.3 (IQR: 3.2–9.8)
4	Uttervall (2024) (31)	Observational, retrospective	MM	Anti-BCMA	58	15.1
5	Mohan (2024) (32)	Observational, retrospective	MM	Anti- BCMA (teclistamab)	110	3.5 (range: 0.4–10.9)
6	Jourdes (2024) (5)	Observational, retrospective	MM	Anti-BCMA (87%) (teclistamab [67%], elranatamab [13%]), anti- GPRC5D (talquetamab [13%])	229	7 (IQR: 4–12)
7	Nooka (2024) (16)	Clinical trial subanalysis	MM	Anti-BCMA (teclistamab)	165	22.8 (IQR: 0.3–33.6)
8	Lancman G (2023) (3)	Observational, retrospective	MM	Anti-BCMA	37	18.6
9	Hammons (2024) (33)	Observational, retrospective	MM	Anti-BCMA (69%); anti-GPRC5D (31%) (monotherapy [15.5%], combination with daratumumab or pomalidomide [15.5%])	96 BsAbs courses in 90 patients	Anti-BCMA: 5.3 (range 0.9–26.1); anti-GPRC5D: 5.6 (range 3.0–19.4); combination: 7.4 (range 1.4–11.0)
10	Bannerji (2022) (11)	RCT	B-cell lymphoma	Anti-CD20 (odronextamab)	145	4.2 (IQR: 1.5–11.5)
11	Kantarjian (2017) (8)	RCT	B-ALL	Anti-CD19 (blinatumomab)	271	11.7
12	Foà (2020) (25)	RCT	B-ALL	Anti-CD19 (blinatumomab)	63	18 (range: 1–25)
13	Thieblemont (2023) (9)	RCT	B-cell lymphoma	Anti-CD20 (epcoritamab)	157	10.7
14	Matasar (2024) (10)	RCT	B-cell lymphoma	Anti-CD20 (mosunetuzumab)	218	14 (range: 0–28)
15	Budde (2024) (26)	RCT	B-cell lymphoma	Anti-CD20 (mosunetuzumab)	120	Phase 1: 41.5; phase 2: 23.9
16	Cani (2025) (34)	Observational, retrospective	MM	Anti-BCMA (64%) (teclistamab [48.7%], elranatamab [15.2%]); anti-GPRC5D (36% [elranatamab])	158^a^	Anti-BCMA: 6.1 (IQR: 2.0–12.0); anti-GPRC5D: 4.5 (IQR: 2.0–7.0)
17	Sim (2023) (35)	Observational, retrospective	MM	Anti-BCMA (31%); anti-GPRC5D (69%)	39	5.1 (range: 1.7–9.1)
18	Pei (2025) (27)	Observational, retrospective	MM	Anti-BCMA (teclistamab)	23	5.8 (range: 0.2–15.9)
19	Bahlis (2023) (14)	RCT	MM	Anti-BCMA (elranatamab)	101	12.0
20	Bahlis (2022) (24)	RCT	MM	Anti-BCMA (elranatamab)	123	14.7 (range: 0.2–25.1)
21	Bar (2023) (23)	RCT (abstract communication)	MM	Anti-BCMA (alnuctamab)	73	7.4 (range: 0.5–19.9)
22	Chari (2022) (15)	RCT	MM	Anti-GPRC5D (talquetamab)	232	SC 405 μg dosing: 11.7 (range: 1.0–21.2); SC 800 μg dosing: 4.2 (range: 0.7–13.7); IV dosing: 4.0 (range: 0.4–40.5)
23	Han (2025) (36)	Observational, retrospective	MM and B-cell lymphoma	NR	103	NR
24	Present single-center cohort (2026)	Observational, retrospective	MM	Anti-BCMA (74.5%); anti-GPRC5D (23.5%); anti-FcRH5 (1.0%); combination (anti-BCMA + anti-GPRC5D [1.0%])	98	10.4 (IQR 2.8–18.6)

B-ALL, B-cell precursor acute lymphoblastic leukemia; BCMA, B-cell maturation antigen; BsAbs, bispecific antibody; FcRH5, Fc receptor-homolog 5; GPRC5D, G-protein coupled receptor family C group 5 member D; IQR, interquartile range; IV, intravenous; MM, multiple myeloma; NR, not reported; RCT, randomized controlled trial; SC, subcutaneous.

^a^ Data on CMV events available only for 126 patients.

The total number of patients included was 2,944 (receiving 2,956 courses of BsAb therapy) with a median follow-up of 10.4 months (IQR: 5.8–14.7). The main indication was MM [70.8% (17/24)] (3, 5, 13–16, 23, 24, 27, 28, 30–35), followed by B-cell lymphoma [16.7% (4/24)] (9–11, 26) and B-ALL [8.3% (2/24)] (8, 25). One single study included mixed data from MM and B-cell lymphoma [4.2% (1/24)] (36). Monotherapy with a single agent was reported in 58.3% (14/24) of studies (8–11, 13–16, 23–27, 32), whereas 41.7% (10/24) described outcomes for more than one agent or did not provide separate data for individual BsAbs (all of them in MM patients) (3, 5, 28, 30, 31, 33–36).

The BsAbs used by order of frequency were as follows: mixed anti-BCMA and anti-GPRC5D [20.8% (5/24)] (5, 28, 33–35), mixed anti-BCMA [12.5% (3/24)] (3, 30, 31), teclistamab [12.5% (3/24)] (16, 27, 32), blinatumomab [8.3% (2/24)] (8, 25), elranatamab [8.3% (2/24)] (14, 24), mosunetuzumab [8.3% (2/24)] (10, 26), epcoritamab [4.2% (1/24)] (9), alnuctamab [4.2% (1/24)] (23), linvoseltamab [4.2% (1/24)] (13), odronextamab [4.2% (1/24)] (11), talquetamab [4.2% (1/24)] (15), and mixed anti-BCMA, anti-GPRC5D, and anti-FcRH5 BsABs [4.2% (1/24], our single-center cohort]. One study did not report details on the type of BsAbs [4.2% (1/24)] (36). No studies including patients receiving etentamig or glofitamab were eligible.

The routine use of antiviral prophylaxis against VZV (acyclovir and/or valacyclovir) was reported in 58.3% (14/24) of the studies (3, 5, 9, 13, 16, 24, 25, 28, 31–35). Explicit information on the use of antiviral prophylaxis against CMV (valganciclovir and/or letermovir) was provided in 45.8% (11/24) of the studies. Most of them [81.2% (9/11)] stated that no patient received anti-CMV prophylaxis (5, 9, 11, 16, 27, 28, 33, 34, 36), and only two [18.2% (2/11)] reported marginal use rates. One study detailed the use of prophylaxis in 3% of the patients (35), whereas secondary prophylaxis after a previous episode of CMV disease was administered in one course of BsAb therapy (1.0%) in our single-center cohort (**Supplementary Table 2**).

#### 3.2.3 CMV infection outcomes

All the 24 studies reported data for at least one of the study outcomes. In detail, data of the frequency of csCMVi was provided in 19 studies (2,362 patients) (3, 8–11, 13–16, 23–25, 27, 28, 30, 31, 34, 36), data on any CMV DNAemia in 23 studies (2,846 patients) (3, 5, 8–11, 13, 15, 16, 23, 24, 26–28, 30, 31, 33–36), and data on CMV disease in 17 studies (1,912 patients) (3, 10, 11, 13–15, 23, 24, 27, 28, 30–34, 36).

**Figure 2:**
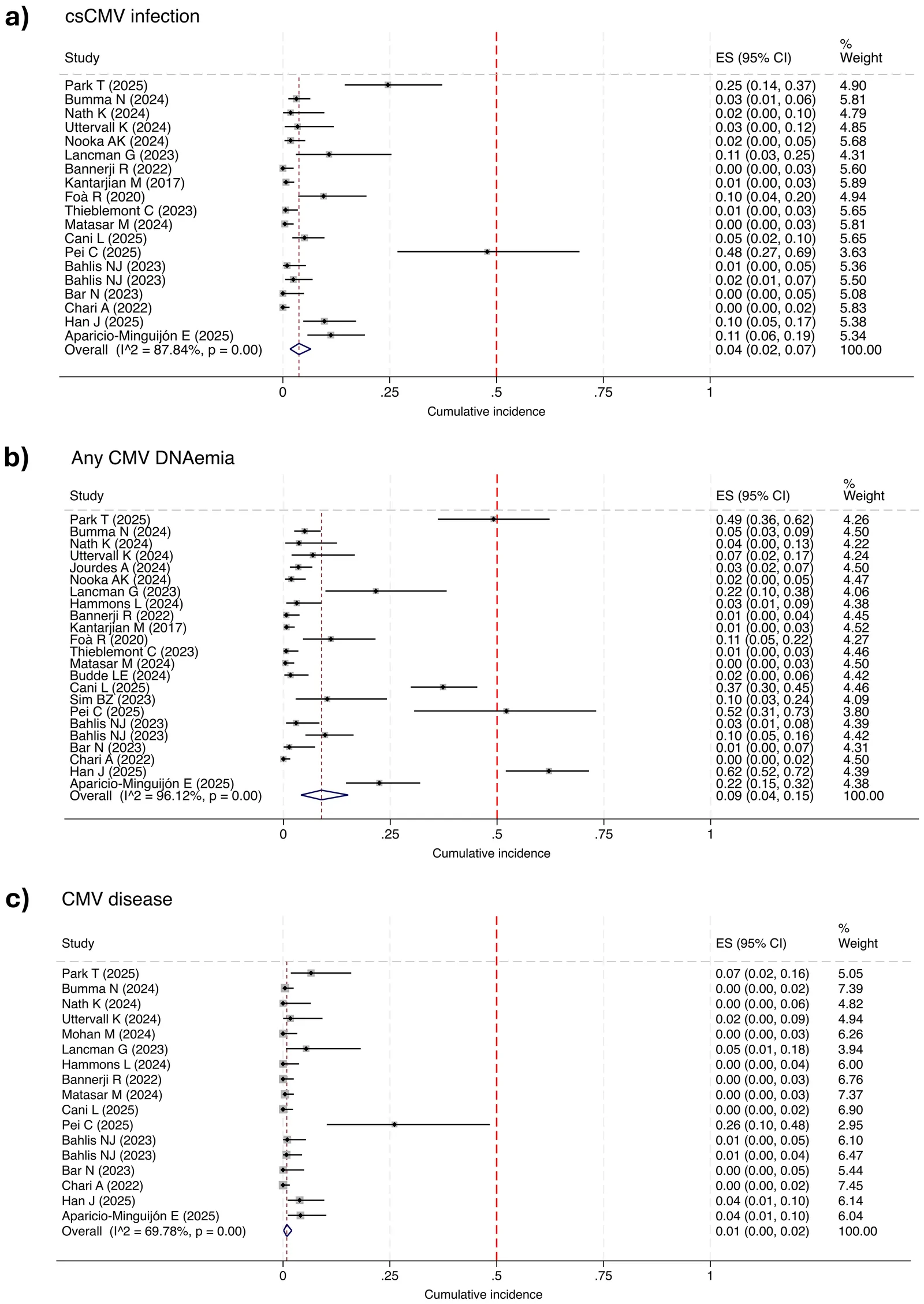
Forest plot of cumulative incidence of **(a)** csCMVi, **(b)** any CMV DNAemia, and **(c)** CMV disease in the entire set of studies included in the meta-analysis. CI, confidence interval; CMV, cytomegalovirus; csCMVi, clinically significant cytomegalovirus infection.

The pooled cumulative incidence rate of csCMVi was 4% (95% CI: 2%–7%; high heterogeneity (I^2^ 87.84%; *P*-value <0.001)]. The pooled cumulative incidence rate of any CMV DNAemia was 9% [95% CI: 4%–15%; high heterogeneity (I^2^ 96.12%; *P*-value <0.001)]. Finally, the pooled cumulative incidence rate of CMV disease was 1% [95% CI: 0%–2%; moderate heterogeneity (I^2^ 69.74%; *P*-value <0.001)]. The corresponding forest plots and funnel plots are shown in **Figure 2**.

The visual inspection of funnel plots for the three estimates revealed evidence of asymmetry suggestive of publication bias in favor of studies with larger prevalence rates (**Supplementary Figure 1**). The asymmetry was confirmed by the Egger test *(P-*values <0.005).

#### 3.2.4 Sensitivity analyses

##### 3.2.4.1 CMV infection by underlying disease

Regarding the underlying malignancy, 17 studies (70.8%) addressed therapies for MM (3, 5, 13–16, 23, 24, 27, 28, 30–35) and 6 studies (25.0%) for B-ALL or B-cell lymphoma (8–11, 25, 26), and one study (4.2%) included both MM and B-cell malignancy patients (36). In studies on MM, the pooled cumulative incidence rate of csCMVi was 5% [95% CI: 2%–9%; high heterogeneity (I^2^ 88.59%; *P*-value <0.001)]. The pooled cumulative incidence rate of any CMV DNAemia was 10% [95% CI: 5%–18%; high heterogeneity (I^2^ 94.93%; *P*-value <0.001)], whereas the estimate for CMV disease was 1% [95% CI: 0%–2%; moderate heterogeneity (I^2^ 71.75%; *P*-value <0.001)].

**Figure 3:**
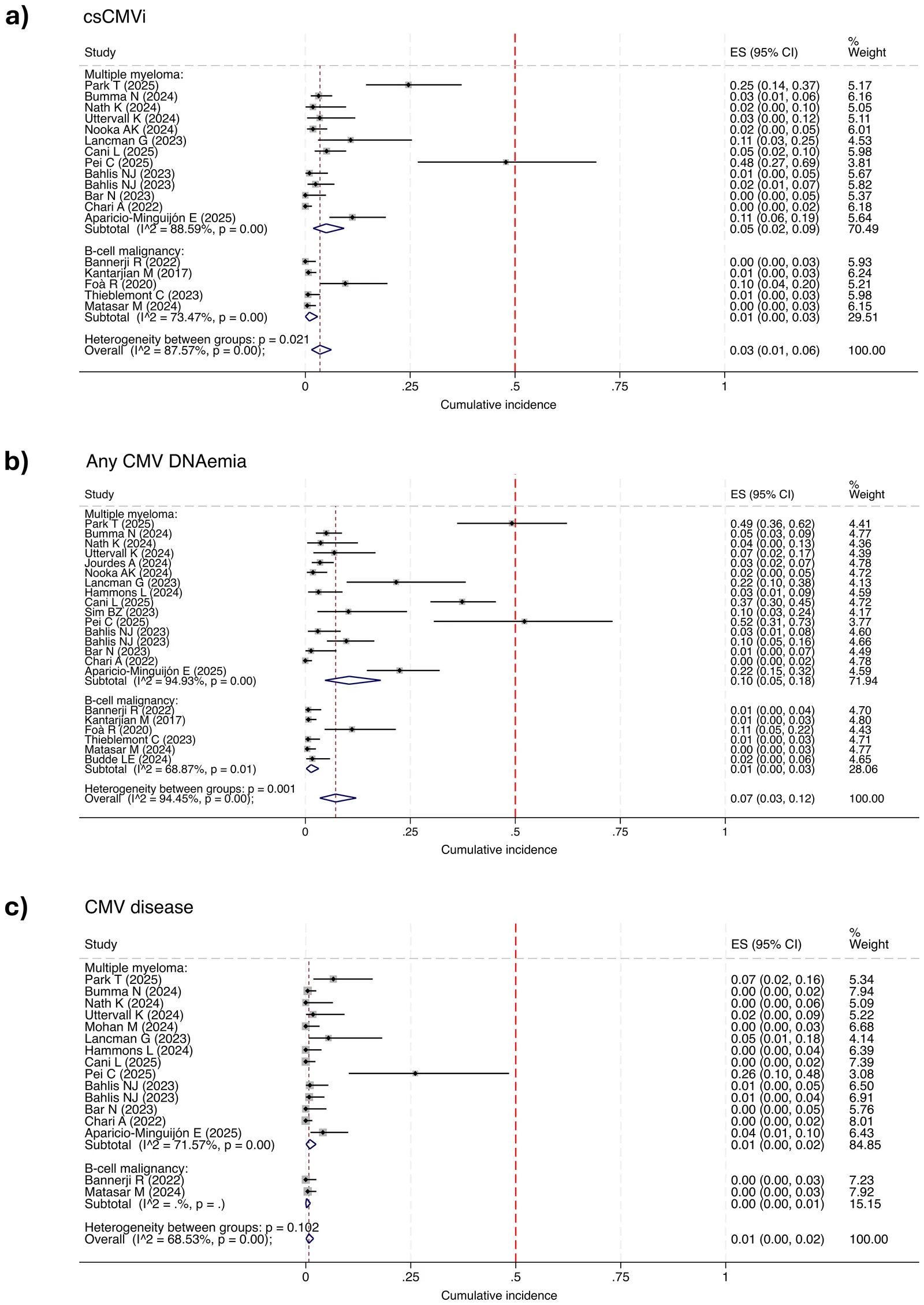
Forest plot of cumulative incidence of **(a)** csCMVi, **(b)** any CMV DNAemia, and **(c)** CMV disease according to the underlying hematological malignancy. CI, confidence interval; CMV, cytomegalovirus; csCMVi, clinically significant cytomegalovirus infection.

In studies on B-cell malignancies, the pooled cumulative incidence rate of csCMVi was 1% [95% CI: 0%–37%; moderate heterogeneity (I^2^ 73.47%; *P*-value <0.001)]. The cumulative incidence rate of any CMV DNAemia was 1% [95% CI: 0%–3%; moderate heterogeneity (I^2^ 68.87%; *P*-value <0.001)], and the pooled cumulative of CMV disease was 0% (95% CI: 0%–1%; non-evaluable heterogeneity). Forest plots are shown in **Figure 3**.

The study by Han et al. did not provide separate data according to the underlying disease and overall reported higher aggregated frequencies of csCMVi (15.5%), CMV DNAemia (62.1%), and CMV disease (3.9%) (36).

##### 3.2.4.2 CMV infection by frequency of CMV DNAemia monitoring

Four studies (16.7%) (3, 26, 27, 34) reported that DNAemia was monitored by qPCR at a regular basis during the course of BsAb therapy. In the remaining 20 studies (83.3%), monitoring was not systematically performed or data about monitoring practices were not available. In the pooled analysis of studies without routine monitoring, the cumulative incidence rate of csCMVi was 3% [95% CI: 1%–5%; high heterogeneity (I^2^ 85.50%; *P*-value <0.001)], the cumulative incidence rate of any CMV DNAemia was 6% [95% CI: 3%–12%; high heterogeneity (I^2^ 95.34%; *P*-value <0.001)], and the cumulative incidence rate of CMV disease was 1% [95% CI: 0%–1%; moderate heterogeneity (I^2^ 53.49%; *P*-value = 0.01)].

**Figure 4:**
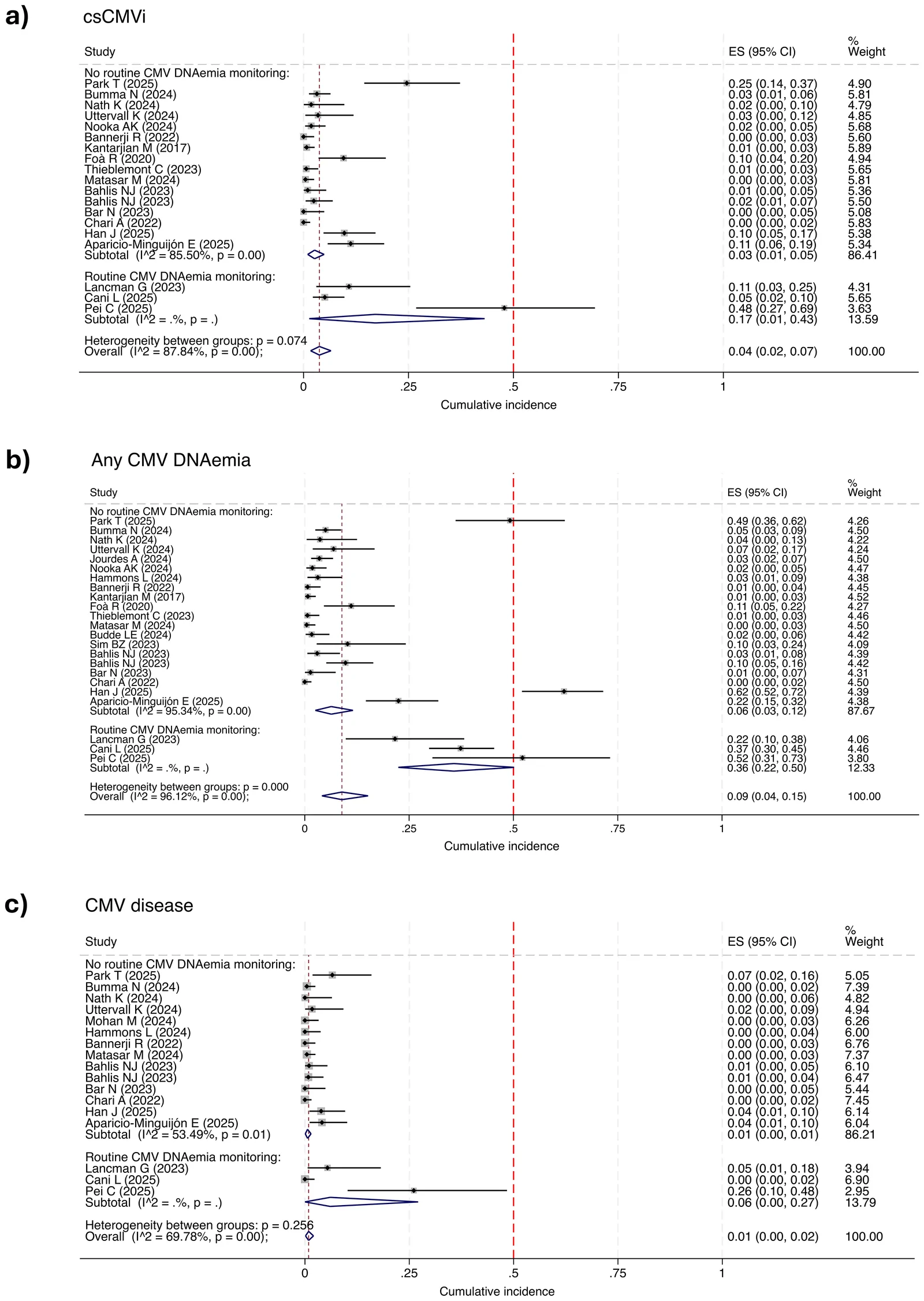
Forest plot of cumulative incidence of **(a)** csCMVi, **(b)** any CMV DNAemia, and **(c)** CMV disease according to the frequency of CMV DNAemia monitoring by qPCR. CI, confidence interval; CMV, cytomegalovirus; csCMVi, clinically significant cytomegalovirus infection.

In studies with regular CMV DNAemia monitoring, the pooled cumulative incidence rates of csCMVi, any CMV DNAemia, and CMV disease were 17% [95% CI: 1%–43%], 36% [95% CI: 22%–50%], and 6% [95% CI: 0%–27%], respectively. Heterogeneity was not evaluable for any of these estimates. Forest plots are shown in **Figure 4**.

##### 3.2.4.3 CMV infection by target of BsAbs

**Figure 5:**
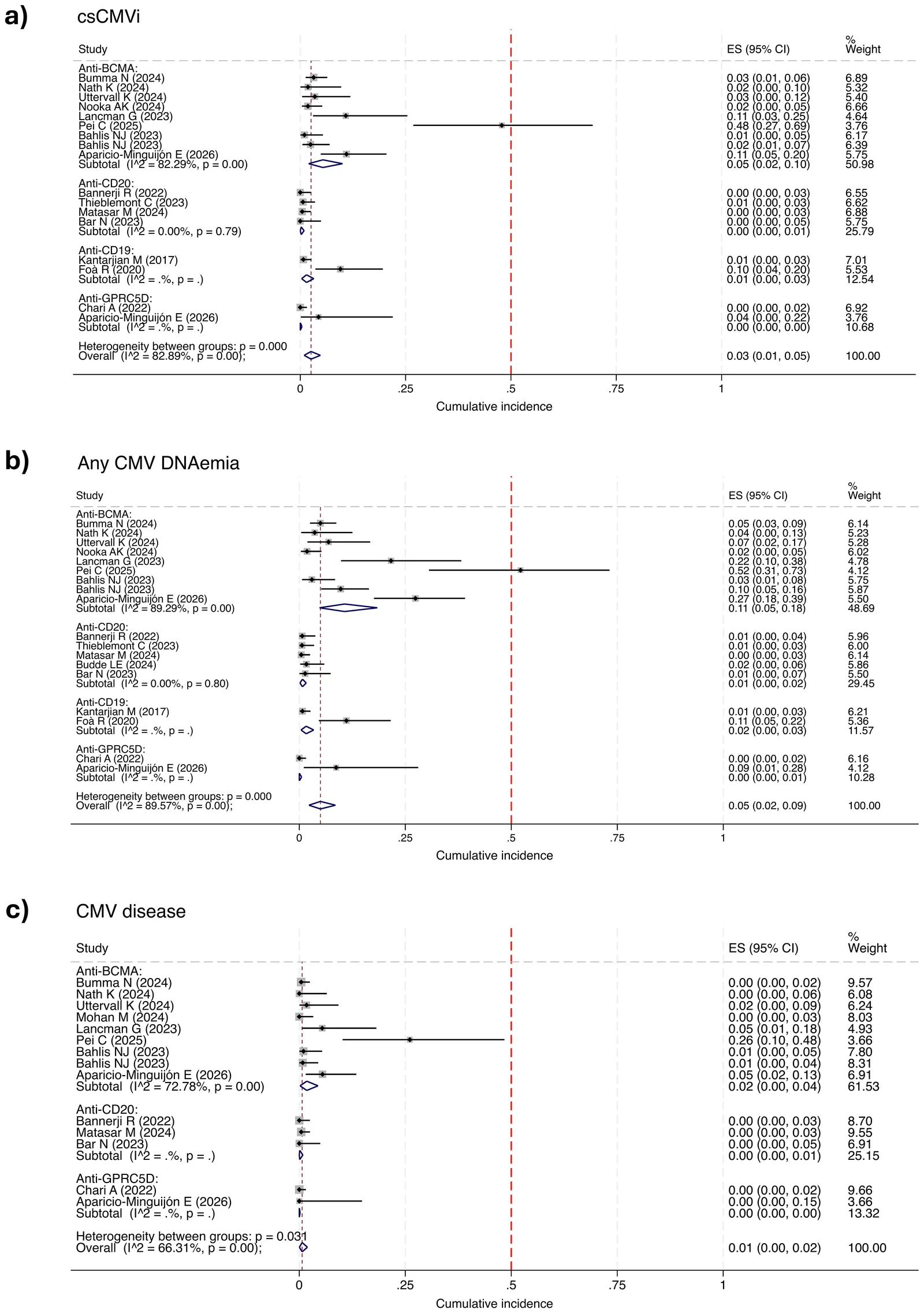
Forest plot of cumulative incidence of **(a)** csCMVi, **(b)** any CMV DNAemia, and **(c)** CMV disease according to target of BsAbs. BCMA, B-cell maturation antigen; FcRH5, Fc receptor-homolog 5; GPRC5D, G-protein coupled receptor family C group 5 member D; CI, confidence interval; CMV, cytomegalovirus; csCMVi, clinically significant cytomegalovirus infection.

We also analyzed the occurrence of CMV events according to the type of BsAbs (**Figure 5**). After excluding studies that reported the mixed use of agents with different specificities, 19 studies were pooled in this sensitivity analysis: nine (47.4%) with anti-BCMA BsAbs (3, 14, 16, 24, 27, 30–32), two (10.5%) with anti-GPRC5D BsAbs (15), five (26.3%) with anti-CD20 BsAbs (9–11, 23, 26), and two (10.5%) with anti-CD19 BsAbs (8, 25). No studies other than our single-center cohort included patients treated with anti-FcRH5 BsAbs, thus precluding a pooled analysis.

For anti-BCMA BsAbs, the cumulative incidence rate of csCMVi was 5% [95% CI: 2%–10%; high heterogeneity (I^2^ 82.29%; *P*-value <0.001)], the cumulative incidence rate of any CMV DNAemia was 11% [95% CI: 5%–18%; high heterogeneity (I^2^ 82.29%; *P*-value <0.001)], and the cumulative incidence rate of CMV disease was 2% [95% CI: 0%–4%; moderate heterogeneity (I^2^ 72.78%; *P*-value <0.001)].

For anti-GPRC5D BsAbs, the cumulative incidence rates of csCMVi, CMV DNAemia, and CMV disease had a common estimate of 0% (95% CI: 0%–0%; non-evaluable heterogeneity).

Regarding anti-CD19 BsAbs, the cumulative incidence rate of csCMVi and any CMV DNAemia was 1% (95% CI: 0%–3%; non-evaluable heterogeneity) and 2% (95% CI: 0%–3%; non-evaluable heterogeneity), respectively. No episodes of CMV disease were reported.

For anti-CD20 BsAbs, the cumulative incidence rate of csCMVi was 0% [95% CI: 0%–1%; low heterogeneity (I^2^ 0.00%; *P*-value = 0.79)], the cumulative incidence rate of any CMV DNAemia was 1% [95% CI: 0%–2%; low heterogeneity (I^2^ 0.00%; *P*-value = 0.80)], and the cumulative incidence rate of CMV disease was 0% [95% CI: 0%–1%; non-evaluable heterogeneity].

#### 3.2.5 Quality evaluation

**Table 4** shows the NOS quality assessment according of the 12 observational studies, including our single-center cohort (3, 5, 27–35). All studies were categorized as moderate quality with 5 or 6 stars. The main limitation was the absence of clear definitions for CMV infection and the low quality of information on the main outcomes.

**Table 4:** Quality evaluation of the observational cohort studies according to the NOS scale.

Study, year	NOS quality indicators			
	Selection^a^	Comparability^b^	Outcome	Total score
Park (2025) (28)	***	Not applicable	***	6
Nath (2024) (30)	***	Not applicable	**	5
Uttervall (2024) (31)	***	Not applicable	**	5
Mohan (2024) (32)	***	Not applicable	**	5
Jourdes (2024) (5)	***	Not applicable	**	5
Lancman (2023) (3)	***	Not applicable	***	6
Hammons (2024) (33)	***	Not applicable	**	5
Cani (2025) (34)	***	Not applicable	***	6
Sim (2023) (35)	***	Not applicable	**	5
Pei (2025) (27)	***	Not applicable	***	6
Han (2025) (36)	***	Not applicable	**	5
Present single-center cohort (2026)	***	Not applicable	***	6

NOS, Newcastle–Ottawa Quality Assessment Scale.

^a^ One out of the four evaluable items of the “Selection” dominion was not applicable due to the absence of comparator group.

^b^ Not applicable due to the absence of comparator group.

** indicates 2 points and *** indicates 3 points. The sum of these points of each section (column) is represented in the last column (total score).

## 4 Discussion

The present meta-analysis explores the landscape of CMV infection following BsAb therapy. Our findings demonstrate a low—although still relevant—frequency of CMV events, with a pooled cumulative incidence rate of 4% for csCMVi and 9% for any CMV DNAemia. Notably, the occurrence of clinically evident CMV disease was negligible (1%). Risk profiles varied depending on the type of BsAb and the underlying malignancy. Overall, MM patients appeared to be at a higher risk than those with B-cell lymphoma or B-ALL. This discrepancy may be explained by baseline clinical characteristics, since MM patients are typically older and have a higher cumulative burden of immunosuppression (37). In contrast, we found a low incidence of csCMVi or CMV DNAemia in patients treated with anti-CD20 or anti-CD19 BsAbs, and no reported cases of CMV disease. The only point of divergence was a phase 2 RCT of dasatinib plus corticosteroids followed by blinatumomab as the first-line therapy for B-ALL, which found a cumulative incidence rate of grade ≥2 CMV infection of 11% (25). Since the ABL tyrosine kinase inhibitor dasatinib has been associated with an increased risk of CMV disease due to off-target effects (38), the specific contribution of blinatumomab is uncertain.

The pooled analysis of studies focused on MM showed a cumulative incidence rate of 5% for csCMVi and 1% for CMV disease, although these estimates should be interpreted with caution because of the moderate to high between-study heterogeneity observed. The persistence of I² values consistently exceeding 70% across the different subgroup analyses suggests that, even after stratifying by certain characteristics—such as the underlying disease, frequency of monitoring, or type of BsAb—substantial between-study differences remain with respect to study design (RCT versus observational study), sample size, antiviral prophylaxis practices, definitions of CMV events, and follow-up. In addition, the 95% CIs reported by some individual studies (27) were particularly wide, reflecting the uncertainty derived from small sample sizes. The use of anti-BCMA BsAbs appeared to be associated with an increased rate of CMV reactivation compared with talquetamab. This finding aligns with previous studies reporting higher risk of overall infection—not restricted to CMV—with anti-BCMA agents (33). For instance, Cani et al. found a 5-month cumulative incidence rate of any-grade infection of 38.6% in patients with relapsed/refractory MM treated with anti-BCMA BsAbs, as compared with 28.1% with anti-GPRC5D BsAbs (34).

A key finding of the present study was that the estimated risk of CMV events was substantially influenced by the implementation of systematic qPCR surveillance, with markedly different event rates reported by studies that did and did not perform routine CMV DNAemia monitoring. In studies with regular monitoring—all of which included MM patients on anti-BCMA BsAbs (3, 26, 27, 34)—the incidence rates of csCMVi and CMV disease reached 17% and 6%, respectively. The clinical implications of asymptomatic and/or low-level CMV DNAemia remain to be elucidated, in particular in terms of virus-driven accelerated immunosenescence and T-cell exhaustion, as also hypothesized for allo-TPH recipients (27, 39). Consistent with this notion, Pei et al. found that patients who experienced CMV replication had a higher incidence of concurrent infections, even after viral clearance, as well as lower response rates and poorer survival (27). In our cohort, we observed a higher all-cause mortality and a lower MM relapse rate in patients that experienced csCMVi, although the interpretation of these findings may be confounded due to the lack of multivariable adjustment and competing risk analysis. Uncertainty extends to the CMV DNAemia thresholds for initiating preemptive antiviral therapy, which vary significantly across centers. Nevertheless, while studies lacking systematic monitoring may have underestimated the rate of csCMVi, it is unlikely that cases of clinically overt disease would have been missed. Therefore, the overall frequency of CMV disease appears to be low regardless of the monitoring strategy applied.

In the related field of CAR-T therapy, a recent systematic review (which ultimately included only four studies) found that approximately one-fourth of patients experienced CMV infection. Although non-Hodgkin lymphoma accounted for 80% of the study population, MM was relatively more common among patients that developed CMV events (6). This work shared many of the limitations of our own systematic review, including heterogeneity in CMV monitoring protocols, duration of follow-up, assessment of baseline serostatus, and CMV prevention and treatment strategies. Interestingly, some authors have reported worse outcomes—in terms of higher one-year mortality and rates of disease progression and relapse—among CAR-T recipients with CMV reactivation (40, 41), which would align with the hypothesis of CMV-associated T-cell exhaustion.

The most common form of CMV disease in our pooled analysis was pneumonitis (38.1% [8/21]), followed by viral syndrome (23.8% [5/21]). Notably, all the episodes of viral syndrome were reported from a single study that implemented systematic DNAemia monitoring (27). From a clinical perspective, the occurrence of viral syndrome may have been over- or underestimated, as patients treated with BsAbs frequently experience febrile episodes attributable to other causes. On the other hand, the diagnostic criteria for CMV viral syndrome have not been well established for the hematological setting (19).

Previous studies have identified a number of risk factors for CMV infection, including older age, the occurrence of CMV DNAemia before the initiation of BsAb therapy, or the concurrent use of anti-CD38 agents (28, 36). Our single-center cohort identified additional factors in MM patients—poor ECOG performance status, grade ≥3 CRS, prior allo-HSCT, and profound neutropenia—which overall point to the relevance of the net state of immunosuppression. As with the systematic literature review, the absence of an institutional protocol for CMV DNAemia monitoring, preemptive antiviral therapy, and the use of letermovir as secondary prophylaxis limits the ability to draw firm conclusions from our experience.

In light of the data reviewed, we consider that the frequency of csCMVi and CMV disease is overall low, particularly for patients receiving anti-CD19 or anti-CD20 BsAbs for B-cell malignancies or anti-GPRC5D BsAbs for MM. In these populations, it is reasonable to assume that systematic DNAemia monitoring and antiviral prophylaxis are unlikely to provide substantial clinical benefit. Conversely, patients treated with anti-BCMA BsAbs would face a higher risk of csCMVi—although estimates vary according to the frequency of monitoring—while the occurrence of disease remains uniformly uncommon. Routine qPCR assessment may be reasonable in the presence of additional risk factors (i.e., previous allo-HSCT or severe CRS). Pending results from future intervention studies, we conclude that universal prophylaxis is not justified even in this high-risk subgroup.

Our research has various limitations. The main constraint is the lack of granular data and the incomplete reporting on CMV events, as reflected by moderate NOS quality scores. Data on the definitions applied for CMV infection and the use of antiviral prophylaxis were scarce. Notably, details on the baseline CMV serostatus was available for only 47 out of 92 BsAb courses in our cohort and was virtually absent across the studies included in the meta-analysis. This limitation is particularly relevant, as distinguishing CMV reactivation from primary infection has important implications for clinical management and preventive strategies. Future trials and observational studies should systematically assess the baseline CMV serostatus of enrolled patients. We detected potential publication bias toward studies reporting higher prevalences. As discussed above, the clinical significance of the high rates of CMV DNAemia in studies with routine monitoring remains unclear. Finally, while our single-center cohort provides high-quality data, the small number of events limits the statistical power required for multivariable adjustment. Therefore, it is important to emphasize that the unadjusted associations observed for ECOG performance status, severe CRS, prior allo-HSCT, and neutropenia should be interpreted with caution and regarded as merely hypothesis-generating rather than confirmatory.

In conclusion, based on the limited evidence currently available, CMV infection appears to be an uncommon complication in patients receiving anti-CD20 or anti-CD19 BsAbs for B-ALL or B-cell lymphoma. In contrast, it remains a more relevant concern among MM patients on anti-BCMA therapy, although additional data are needed to better define its true incidence and clinical significance. Routine CMV DNAemia monitoring may be considered for high-risk subgroups, particularly those with a prior history of CMV reactivation or additional risk factors. Future studies are needed to refine risk stratification and to identify specific populations that would benefit from targeted surveillance or antiviral prophylaxis.

## Data Availability

The raw data supporting the conclusions of this article will be made available by the authors, without undue reservation.
