# Supplementary material for: Cytomegalovirus infection in patients receiving bispecific antibodies for multiple myeloma and B-cell malignancies: a single-center cohort and meta-analysis
Source: Front Oncol. 2026 Aug 26;16:1918863. doi: 10.3389/fonc.2026.1918863 (PMC13553244; doi:10.3389/fonc.2026.1918863)
Supplement: Supplementary file 1 [file DataSheet1.docx]

**Supplementary Methods**

**Single-center cohort**

*Study definitions*

*Cytomegalovirus (CMV) DNAemia blip* was defined as a single low-level positive CMV qPCR result in a patient receiving prophylaxis with letermovir, with both the preceding and subsequent tests performed at weekly intervals remaining negative. *CMV disease* included both end-organ disease and viral syndrome. *Viral syndrome* was defined by the presence of detectable CMV DNAemia plus fever plus at least one of the following criteria: leukopenia (white blood cell [WBC] count <3.50 x 10^3^ cells/μL if baseline WBC count was ≥4.00 x 10^3^ cells/μL or a decrease >20% if baseline WBC count was <4.00 x 10^3^ cells/μL); atypical lymphocytosis (≥5%); thrombocytopenia (platelet count <100 x 10^3^ cells/μL if baseline count was ≥115 x 10^3^ cells/μL or a decrease >20% if baseline platelet count was <115 x 10^3^ cells/μL); or elevation of ALT or AST of more than 2 times the upper limit of normal. To qualify as CMV viral syndrome, alternative causes of febrile illness (e.g., bacterial infections, respiratory viral infections, CAR-T cell infusion, blood product transfusions, engraftment syndrome, drug-induced fever, acute graft versus host disease or cytokine release syndrome [CRS]) should have been reasonably ruled out. *End-organ disease* included probable or proven diagnostic categories, with the latter requiring the documentation of CMV replication tissue specimens from the relevant organ (e.g., histopathology, virus isolation, immunohistochemistry, or nucleic acid hybridization) in the presence of compatible clinical manifestations [1]. Hypogammaglobulinemia was defined by IgG level <400 mg/dL once the monoclonal component secondary to MM had been subtracted.

*Grading and management of CRS and ICANS*

Severity of CRS was graded as follows: *grade 1* denoted fever with or without constitutional symptoms; *grade 2* denoted hypotension responding to fluids or hypoxia responding to fraction of inspired oxygen (FiO_2_) <0.40; *grade 3* denoted hypotension or hypoxia that required pressors and FiO_2_ >0.40; *grade 4* denoted life-treating symptoms requiring intensive care unit admission. Immune effector cell-associated neurotoxicity syndrome (ICANS) severity was also graded as follows: *grade 1* denoted mild confusion that limits instrumental activities of daily living (ADLs); *grade 2* denoted somnolence that responds to voice and/or moderate confusion that limit ADLs; *grade 3* denoted somnolence that responds to tactile stimuli and/or occurrence of generalized seizure; and *grade 4* denoted unconsciousness difficult to arouse, coma, motor disturbances and/or prolonged seizures [2].

The management of CRS includes the use of antipyretics and tocilizumab for grade 1-2 episodes. Dexamethasone was added in cases of refractoriness to first-line treatment. In severe cases, the use of anakinra or other immunosuppressive agents is considered. The approach to ICANS included the administration of dexamethasone from grade 1 episodes, associated to anakinra or other immunosuppressive agents in case of no improvement or worsening of clinical manifestations [3].

*Prophylaxis regimens and pre-emptive strategies*

Prophylaxis against *Pneumocystis jirovecii* pneumonia was administered throughout the course of BsAbs therapy with double-strength trimethoprim-sulfamethoxazole (TMP-SMX) (160/800 mg three times weekly) or, in cases of TMP-SMX intolerance or severe cytopenias, monthly intravenous (IV) pentamidine (300 mg).

Cytomegalovirus (CMV) DNAemia was not routinely monitored and was assessed at the treating physician’s discretion. Treatment with IV ganciclovir (GCV) (5 mg/kg every 12 hours) or oral valganciclovir (VGCV) (900 mg every 12 hours) for at least two weeks, or oral maribavir (400 mg/12 hours) in selected cases, was initiated in the presence of significant or rapidly increasing CMV DNAemia according to the hematologist’s criteria, or symptomatic disease. Doses were adjusted according to renal function when necessary. All patients were prescribed universal herpes simplex virus prophylaxis with acyclovir (800 mg every 12 hours).

Serum immunoglobulin levels were monitored at monthly intervals. Patients with severe hypogammaglobulinemia (IgG <400 mg/dL) received monthly IV immunoglobulin replacement (IVIG) therapy until levels were confirmed to be ≥400 mg/dL at various points. Selected patients with IgG levels >400 mg/dL and repeated episodes of infection were also given IVIG therapy at the discretion of the treating hematologist.

Pre-medication for CRS consisted of acetaminophen, dexchlorpheniramine and dexamethasone during step-up doses. Tocilizumab or other anti-interleukin (IL)-6 agents were not routinely used for prophylaxis.

**Systematic literature review and meta-analysis**

*Search strategy*

The search was performed in PubMed (Medline), Embase and Web of Science databases by using the following combination of terms: (‘Bispecific antibodies’ OR ‘BsAbs’ OR ‘BITE’ OR ‘teclistamab’ OR ‘elranatamab’ OR ‘linvoseltamab’ OR ‘etentamig’ OR ‘ABBV-383’ OR ‘talquetamab’ OR ‘cevostamab’ OR ‘alnuctamab’ OR ‘blinatumomab’ OR ‘glofitamab’ OR ‘mosunetuzumab’ OR ‘odronextamab’ OR ‘epcoritamab’) AND (‘leukemia’ OR ‘multiple myeloma’ OR ‘lymphoma’ OR ‘hematological malignancies’) AND (‘infection’ OR ‘infectious complications’ OR ‘CMV’ OR ‘cytomegalovirus’). Electronic databases were searched from inception to September 23, 2025. The references of the resulting articles as well as excluded recent reviews and meta-analysis were reviewed to identify potentially relevant missed publications.

*Grading of episodes of CMV infection*

The severity of CMV infection was established according to the applicable version of the Common Terminology Criteria for Adverse Events (CTCAE) as follows: *grade 1* denoted asymptomatic or mild infection; *grade 2* denoted moderate symptoms that require medical intervention; *grade 3* denoted severe infection requiring hospitalization or IV therapy; *grade 4* denoted life-threatening infection and urgent intervention indicated; and *grade 5* denoted death [4].

*References*

1. Ljungman P, Chemaly RF, Khawaya F, et al. Consensus definitions of cytomegalovirus (CMV) infection and disease in transplant patients including resistant and refractory CMV for use in clinical trials: 2024 Update from the Transplant Associated Virus Infections Forum. Clin Infect Dis. 2024;79(3):787-794.
2. Lee DW, Santomasso BD, Locke FL, et al. ASTCT consensus grading for cytokine release syndrome and neurologic toxicity associated with immune effector cells. Biol Blood Marrow Transplant. 2019;25:625-638.
3. Lin Y, Qiu L, Usmani S, Joo CW, Costa L, Derman B, et al. Consensus guidelines and recommendations for the management and response assessment of chimeric antigen receptor T-cell therapy in clinical practice for relapsed and refractory multiple myeloma: a report from the International Myeloma Working Group Immunotherapy Committee. Lancet Oncol. 2024;25:e374-e387.
4. National Cancer Institute. Common Terminology Criteria for Adverse Events (CTCAE) Common Terminology Criteria for Adverse Events (CTCAE) v5.0 [Internet]. 2017. Available online at: <https://www.meddra.org/>

**Supplementary Results**

**Table S1.** Univariable analysis of risk factors for the occurrence of clinically significant CMV infection in the single-center cohort (n = 98 courses of BsAbs therapy).

| **Variable** | **Univariable HR (95% CI)** | ***P*-value** |
| --- | --- | --- |
| Age (per one-year increase) | 1.01 (0.94 - 1.08) | 0.861 |
| Male gender | 0.49 (0.12 – 1.96) | 0.296 |
| Underlying MM Ig subtype [n (%)] |  |  |
| IgG | Ref. |  |
| IgA | 3.87 (0.85 – 17.57) | 0.300 |
| Light chain | 0.99 (0.17 – 5.94) |  |
| MM Revised International Staging System |  |  |
| One point | Ref. |  |
| Two points | 1.10 (0.21 – 5.62) | 0.854 |
| Three points | 0.60 (0.06 – 5.81) |  |
| ECOG (per one-point increase) | 2.79 (1.54 – 5.07) | **0.004** |
| Prior lines of treatment (per line) | 1.03 (0.76 – 1.41) | 0.837 |
| Prior anti-CD38 mAb | NE | - |
| Prior antibody-drug conjugates | NE | - |
| Prior BsAbs | 1.65 (0.20 – 13.44) | 0.659 |
| Prior autologous HSCT | 1.42 (0.41 - 4 .87) | 0.578 |
| Prior allogeneic HSCT | 12.16 (1.71 – 86.45) | **0.024** |
| Prior CAR-T | 1.29 (0.16 – 10.42) | 0.816 |
| Concomitant therapy with IMiD | NE | - |
| Concomitant therapy with anti-CD38 mAb | NE | - |
| Nadir grade 4 neutropenia | 3.59 (0.74 – 17.42) | 0.082 |
| Nadir grade 4 lymphopenia | 1.16 (0.24 – 5.62) | 0.848 |
| Grade 4 neutropenia at initiation of BsAbs therapy | 20.0 (3.65 – 109.44) | **0.007** |
| Grade 4 lymphopenia at initiation of BsAbs therapy | NE | - |
| HGG at initiation of BsAbs therapy | NE | - |
| HGG during the course of BsAbs therapy | NE | - |
| CRS (any grade) | 3.63 (0.45 – 29.04) | 0.154 |
| Grade ≥2 | 0.61 (0.07 – 5.05) | 0.213 |
| Grade ≥3 | 19.9 (3.65 – 109.44) | **0.006** |
| ICANS (any grade) | NE | - |
| Steroids for CRS/ICANS | 1.73 (0.43 – 6.98) | 0.456 |
| Tocilizumab for CRS/ICANS | 0.62 (0.13 – 3.03) | 0.539 |
| Anakinra for CRS/ICANS | NE | - |
| Trimethoprim-sulfamethoxazole prophylaxis | NE | - |
| Acyclovir prophylaxis | NE | - |
| Immunoglobulin replacement therapy | 1.10 (0.23 – 5.32) | 0.904 |
| BCMA: B-cell maturation antigen; BsAbs: bispecific antibodies; CAR-T: chimeric antigen receptor T-cell; CRS: cytokine release syndrome; FcRH5: Fc receptor-homolog 5; GPRC5D: G protein-coupled receptor class C group 5 member D; HSCT: hematopoietic stem-cell transplantation; ICANS: immune effector cell-associated neurotoxicity syndrome; IQR: interquartile rank; MM: multiple myeloma; SD: standard deviation.  *NE: Non estimable due to low number of events with unstable coefficients. | | |

**Table S2**. Antiviral prophylaxis and CMV DNAemia monitoring practices and definitions for CMV events in the studies included in the meta-analysis.

| **Study** | **Author and year** | **(Val)acyclovir prophylaxis** | **(Val)ganciclovir or letermovir prophylaxis** | **CMV DNAemia monitoring** | **CMV infection definitions** |
| --- | --- | --- | --- | --- | --- |
| 1 | Park T et al (2025) (25) | Valacyclovir 500 mg once daily | No | NR | CMV reactivation was defined as the detection of CMV replication in blood (or other sites) in CMV-seropositive patients. csCMVi was defined as CMV disease or CMV viremia leading to preemptive treatment. CMV disease was defined as the presence of attributable clinical symptoms and/or signs with the documentation of CMV in tissue from the relevant organ by histopathology, virus isolation, rapid culture, immunohistochemistry, or DNA hybridization. |
| 2 | Bumma N (2024) (15) | Acyclovir or equivalent prophylaxis for all patients | Patients with severe immune dysfunction may warrant CMV prophylaxis in accordance with local institutional guidelines | NR | NR |
| 3 | Nath K (2024) (27) | NR | Prophylaxis was administered according to institutional and protocol guidelines | NR | NR |
| 4 | Uttervall K (2024) (28) | Valacyclovir | Antiviral prophylaxis per local standard of care (87.0%) | NR | NR |
| 5 | Mohan M (2024) (29) | Prophylaxis for HSV/VZV (99%) | Infections prevention and monitoring strategies were administered according to institutional guidelines | NR | NR |
| 6 | Jourdes A (2024) (5) | Acyclovir o valacyclovir (91%) | No | NR | NR |
| 7 | Nooka AK (2024) (13) | Prophylaxis (68.5%) (acyclovir [40%], valacyclovir [29%]) | No | Only in the presence of suspected symptoms or unexplained fever | CMV viremia was reported if previous CMV exposure could not be confirmed |
| 8 | Lancman G (2023) (3) | VZV prophylaxis was universal | No | After two cases of CMV esophagitis were observed, monthly CMV DNAemia monitoring was implemented | NR |
| 9 | Hammons L (2024) (30) | All patients were on antimicrobial prophylaxis with acyclovir or valacyclovir | No | No | NR |
| 10 | Bannerji R (2022) (18) | Prophylaxis as per local institutional guidelines (including antiviral prophylaxis for patients with previous HSV) | Prophylaxis as per local institutional guidelines (including antiviral prophylaxis for patients with previous CMV) | NR | NR |
| 11 | Kantarjian M (2017) (21) | No | High-risk patients (prior CMV re-activation or risk constellation in prior allo-HSCT) were managed by CMV DNAemia monitoring with early therapeutic intervention or anti-CMV prophylaxis | High-risk patients (prior CMV re-activation or risk constellation in prior allo-HSCT) were managed by intensive (biweekly) CMV DNAemia monitoring with early therapeutic intervention or prophylaxis | NR |
| 12 | Foà R (2020) (20) | Acyclovir was given to prevent HSV infection | NR | NR | NR |
| 13 | Thieblemont C (2023) (16) | Antiviral prophylaxis (e.g., acyclovir 400 mg q8h) was mandatory for patients with a history of recurrent HSV infections, herpes infection during previous therapy, neutropenia and/or <200 CD4+ cells/μL | No | NR | NR |
| 14 | Matasar M (2024) (12) | NR | NR | NR | NR |
| 15 | Budde LE (2024) (23) | NR | NR | CMV qPCR at screening, C2D1, and when clinically indicated | NR |
| 16 | Cani L (2025) (31) | The institutional prophylaxis guidelines included antiviral therapy for VZV | No | CMV DNAemia was monitored on day 1 of each cycle. Therapy was initiated on the judgment of the treating physician, considering DNAmia kinetics or the presence of symptoms | CMV DNA detection was defined as the detection of CMV DNA in peripheral blood using quantitative PCR. csCMVi was defined as CMV DNAemia leading to initiation of therapy or meeting criteria for CMV disease. CMV disease required compatible clinical symptoms along with virological and/or histopathological evidence of organ involvement. |
| 17 | Sim B Z (2023) (32) | Valacyclovir 500 mg daily was routinely used for HSV/VZV prophylaxis (valacyclovir [94%], famciclovir [3%]) | Valganciclovir (3%) | NR | NR |
| 18 | Pei C (2025) (24) | NR | No | No routine monitoring strategy was performed but CMV DNAemia was tested based on provider preferences on 23 patients | CMV DNAemia (>500 IU/mL). CMV infection was defined as a documented CMV DNAemia, confirmed by two consecutive determinations, in the presence of clinical suspicion. CMV syndrome was defined as the presence of CMV DNAemia plus at least 2 criteria (fever for ≥ 2 days, new or increased fatigue, leukopenia or neutropenia on 2 separate measurements, thrombocytopenia, or elevation of hepatic aminotransferases). CMV end-organ disease was defined by the presence of specific organ involvement. |
| 19 | Bahlis NJ (2023) (19) | NR | NR | NR | NR |
| 20 | Bahlis NJ (2022) (17) | Patients received prophylaxis per local standard of care (antiviral prophylaxis [87.0%]) | NR | NR | NR |
| 21 | Bar N (2023) (14) | NR | NR | NR | NR |
| 22 | Chari A (2022) (22) | NR | NR | NR | NR |
| 23 | Han J (2025) (33) | NR | No | CMV qPCR monitoring was performed according to the attending physicians | CMV infection was defined as any detection of CMV DNAemia even below the limit of quantification (1.49 log IU/mL) by qPCR performed within 180 days after the last BsAbs treatment. csCMVi was defined as CMV infection requiring antiviral therapy either preemptive or therapeutic. CMV disease was defined as CMV infection with end-organ involvement. |
| 24 | Present single-center cohort (2026) | Acyclovir (100%) | Secondary letermovir prophylaxis (1%) | CMV qPCR monitoring was performed according to the attending physicians | CMV DNAemia was defined as CMV DNA detection by qPCR on peripheral blood samples. csCMVi was defined as either the occurrence of CMV disease or the initiation of anti-CMV preemptive therapy based on prespecified CMV DNAemia threshold. CMV disease included the occurrence of CMV end-organ disease or CMV viral syndrome. CMV end-organ disease included probable or proven categories, with the latter requiring the documentation of CMV replication in tissue specimens by viral culture, immunohistochemistry, histopathology or DNA hybridization, in the presence of compatible manifestations |
| allo-HSCT: allogeneic hematopoietic stem-cell transplantation; BsAbs: bispecific antibodies; CMV: cytomegalovirus; csCMVi: clinically significant CMV infection; HSV: herpes simplex virus; NR: not reported; qPCR: quantitative polymerase chain reaction; VZV: varicella-zoster virus. | | | | | |

**Figure S1**. Funnel plots (with pseudo 95% confidence intervals) for the assessment of publication bias of meta-analyses of **(a)** csCMVi, **(b)** any CMV DNAemia and **(c)** CMV disease. CMV: cytomegalovirus; csCMVi: clinically significant cytomegalovirus infection.

**b)**

**a)**

**c)**
